# Reference intervals for thyroid-stimulating hormone in the adult Lebanese population: Results from the last decade

**DOI:** 10.1016/j.heliyon.2025.e42453

**Published:** 2025-02-03

**Authors:** Elie Naous, Riam Hijazi, Marianne Chalhoub, Nada El Ghorayeb, Marie-Hélène Gannagé-Yared

**Affiliations:** a Department of Endocrinology, Saint Joseph University, Faculty of Medicine, Beirut, Lebanon; b Department of Internal Medicine, St's Elizabeth Medical Center, Tufts Medical School and Boston University Medical School, MA, 02135, USA

**Keywords:** Thyroid stimulating hormone, Indirect reference interval, Gender, Age, Adults, Lebanese

## Abstract

**Background:**

Thyroid Stimulating Hormone (TSH) is the most accurate marker in the assessment of thyroid function. Establishing a reference interval (RI) for TSH is essential for interpreting results of thyroid function tests. TSH RI in the Lebanese population using the indirect method is lacking. The aim of this study is to establish TSH RI according to age and gender, and to assess its trend over the last decade using the indirect method.

**Methods:**

TSH values of 80156 outpatient subjects (age range 18–99) were retrospectively collected between January 2011 and April 2021 from a tertiary university medical center in Beirut. TSH was measured using the same chemiluminescent assay (Immulite 2000).

**Results:**

The median TSH was 1.4 mIU/L (IQ 0.94–2.10). TSH RI was wider compared to the manufacturer one (0.31–4.95 mIU/L vs 0.40–4.0, p < 0.001). There was a decrease in lower reference limit (LRL) and an increase in upper reference limit (URL) with the increase in age categories (p < 0.001 for both comparisons). RI was broader in women compared to men (respectively 0.29–5.20 vs 0.35–4.48 mIU/L, p < 0.001). There are significant differences in the median, LRL and URL across the years and in all age categories (p < 0.001). TSH RI using the indirect method was wider than the direct method one (p < 0.001).

**Conclusion:**

Using the indirect method, TSH RI in the Lebanese population is wider than the manufacturers'one and that those obtained by the direct method and varies according to age, gender and over time. A periodic reevaluation of RI for each population should be done.

## 1 Introduction

Thyroid Stimulating Hormone (TSH) is the most accurate marker of thyroid dysfunction in the absence of pituitary or hypothalamic disease, and it is used as a first-line test in diagnostic algorithms [1]. Reference intervals (RIs) are essential for clinicians to interpret laboratory test results [2]. Reliable RIs are especially important for thyroid hormone assays including TSH because these tests are widely used in the screening, diagnosis, treatment, and monitoring of thyroid disease [3]. The RIs are established by considering the 2.5th and 97.5th percentiles of the values obtained in a reference population [4].

There are two different approaches to define RIs [3]. First, the direct method is based on the recruitment and selection of well-defined reference subjects, followed by collecting and measuring samples to determine the RIs [3]. This method requires strict exclusion criteria and is expensive and time consuming and therefore, it is hard to apply for each laboratory in routine practice. The alternative approach is the indirect method, which is based on the analysis of the available database of a clinical laboratory, and subsequently does not require the collection of samples. This approach has been shown to be simpler, faster, and more cost-effective than direct methods [5,6]. Thus, the International Federation of Clinical Chemistry (IFCC) published recommendations to encourage its use [3]. In addition, the indirect method allows for the determination of RIs tailored to the specific population under study. It utilizes a much larger sample size, yielding findings that are not achievable with the manufacturer's established RIs, which are often based on smaller, less representative populations and variable inclusion/exclusions criteria, leading to narrower RI [7].

RIs for TSH are influenced by different factors such as ethnicity, gender, age, body mass index (BMI), iodine status, smoking, time of day collection, thyroid peroxidase antibodies positivity and inter-assay differences [[8], [9], [10], [11], [12], [13], [14], [15], [16], [17], [18], [19]]. In fact, studies have shown that TSH is higher in females [[8], [9], [10]] and in areas with sufficient iodine intake [11]. In addition, the upper limit of TSH increases with age [9,[12], [13], [14], [15]], BMI [16,17], in the Hispanic population [10,18] and during the morning hours [19]. Therefore, it is important to establish specific RIs for each population [19].

Worldwide studies were conducted to establish RIs for TSH and thyroid hormones using direct [20,21] and indirect methods [9,13,14,22]. In Lebanon, our group was the first one to recently establish RIs of TSH and thyroid hormones in a sample of adult Lebanese individuals using the direct method [23]. However, a comprehensive assessment of RIs using the indirect method in the adult Lebanese population is still lacking.

Moreover, worldwide studies evaluating changes in TSH reference ranges over time are scarce. A change in iodine status was incriminated in these changes [1]. A shift toward the right of the TSH RIs in a sample of the German population was noted after a ten-year follow-up and was likely due to the improved iodine supply in the studied region [11]. Similar studies in the Middle East and more particularly in Lebanon are still missing.

The objective of the current study is to determine, for the first time, the TSH RIs of the Lebanese adult population using the indirect approach and then to evaluate the trend of TSH RIs over the last decade according to age and gender. A comparison of our results with those obtained using the direct method [23] will be subsequently performed.

## 2 Materials and methods

### 2.1 Data collection

TSH values from outpatient individuals were collected from January 1st, 2011, to April 26th, 2021, from the Hospital Information System of Hotel Dieu de France hospital, a tertiary university medical center of Beirut, Lebanon. Subjects aged <18 years, those with a TSH< 0.10 mIU/L or >10 mIU/L and/or those with a repeated TSH value during the same year were excluded from the study to avoid an over-representation of patients with prior evidence of thyroid disease. Subjects' gender, age and physician's specialty were extracted from the medical records. Age categories were divided into 7 categories: 18–30, 31–40, 41–50, 51–60, 61–70, 71–80, and >80 years. Age categories were then further regrouped into 3 categories (<40, 41–70 and > 70 years).

### 2.2 TSH measurement

TSH was measured by a chemiluminescent assay on the Immulite 2000 automate (Siemens, Washington, D.C., USA). The analytic sensitivity of the assay is 0.004 mIU/L and the coefficient of variation of the reaction is less than 6 % for values within the normal range. Reliability of all the measurement results was regularly checked through the assessment of appropriate controls and application of both internal and external quality control principles. There was no change in the assay over the 11-year period.

### 2.3 Ethical considerations

Research have been performed in accordance with the Declaration of Helsinki. The study was approved by the Ethics Committee of Hotel Dieu de France (CEHDF 1524) on the March 23, 2021. No informed consent was requested by our Ethics Committee since the data collection was anonymous and retrospective.

### 2.4 Statistical analysis

The statistical analysis was performed using IBM SPSS (IBM Corp; SPSS Statistics for Windows v28, Armonk, NY, USA). The distribution of TSH was right-skewed. TSH values were expressed as the median with its interquartile range (quartile 1 to quartile 3) and percentiles 2.5 % (p2.5) and 97.5 % (p97.5). Lower reference limit (LRL) was defined as the 2.5th percentile and upper reference limit (URL) as the 97.5th percentile. For the statistical comparisons involving p2.5 and p97.5, TSH was ln-transformed (natural logarithm), giving that ln(TSH) distribution follow a gaussian shape. p2.5 and p97.5 of ln(TSH) were then compared among categories using the independent samples T-test (2 categories) or ANOVA (3 or more categories) with a linearity test for age categories. The medians of TSH were compared using the Mann-Whitney *U* test or the Kruskall-Wallis test, as appropriate. The Chi 2 test was used to compare proportions. A p value < 0,005 was considered as significant.

## 3 Results

### 3.1 RIs in the overall population

**Fig. 1:**
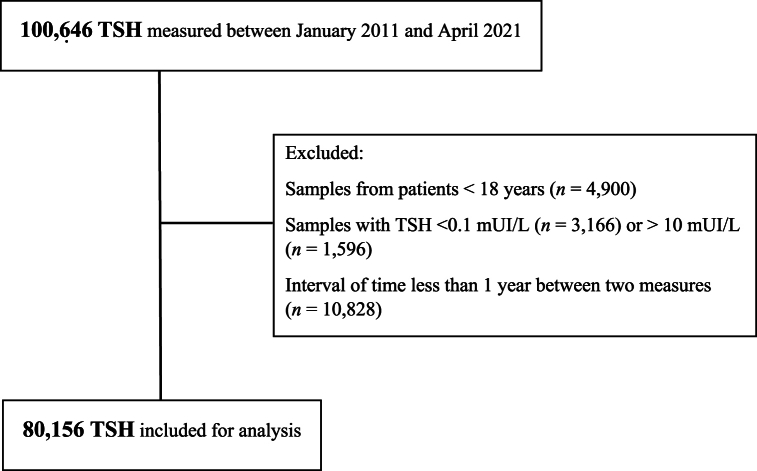
Flow diagram showing the selection criteria for the estimation of TSH reference intervals.

During the study period, TSH was measured in a total of 100 646 out-patient subjects as shown in Fig. 1. Among these, 80 156 samples were included for analysis. As depicted in Table 1, the respective TSH Lower Reference Limit (LRL) and Upper Reference Limit (URL) were 0.31 and 4.95 mIU/L, with a median of 1.40 mIU/L. Compared to the RIs of the manufacturer, the LRL was significantly lower (0.31 vs 0.40 mIU/L), and the URL was significantly higher (4.95 vs 4.0 mIU/L), (p < 0.001 for both comparisons).

**Table 1:** Indirect RIs for TSH according to gender and age categories.

		N (%)	Percentile					P-value		
			2.5	25	50	75	97.5	2.5	50	97.5
**Manufacturer TSH range**			0.40	–	–	–	4.0	<0.001	–	<0.001
**Total TSH**		80156 (100.0)	0.31	0.94	1.40	2.10	4.95			
**Gender**	Women	49733 (62.0)	0.29	0.95	1.45	2.20	5.20	<0.001	<0.001	<0.001
	Men	30423 (38.0)	0.35	0.92	1.37	2.00	4.48			
**Age categories (years)**	18–30	11909 (14.9)	0.44	1.00	1.48	2.10	4.37	<0.001^a^	<0.001^a^	<0.001^a^
	31–40	12363 (15.4)	0.38	1.00	1.41	2.00	4.40			
	41–50	15134 (18.9)	0.33	0.97	1.42	2.10	4.75			
	51–60	15694 (19.6)	0.29	0.90	1.40	2.10	5.10			
	61–70	12177 (15.2)	0.27	0.86	1.36	2.10	5.11			
	71–80	8846 (11.0)	0.23	0.84	1.36	2.11	5.50			
	>80	4033 (5.0)	0.24	0.93	1.50	2.40	6.15			

For the medians, the p-value refers to the Mann-Whitney or the Kruskall-Wallis test, as appropriate; for p2.5 and p97.5, the p-values refer to the T-test or ANOVA, as appropriate.

a Test for linear trend.

### 3.2 RIs according to gender and age

As shown in Table 1, the TSH LRL was lower in women compared to men (0.29 vs 0.35 mIU/L respectively, p < 0.001), while the opposite was found for the URL (5.20 vs 4.48 mIU/L respectively, p < 0.001) and for the median (1.45 vs 1.37 mIU/L, p < 0.001).

**Fig. 2:**
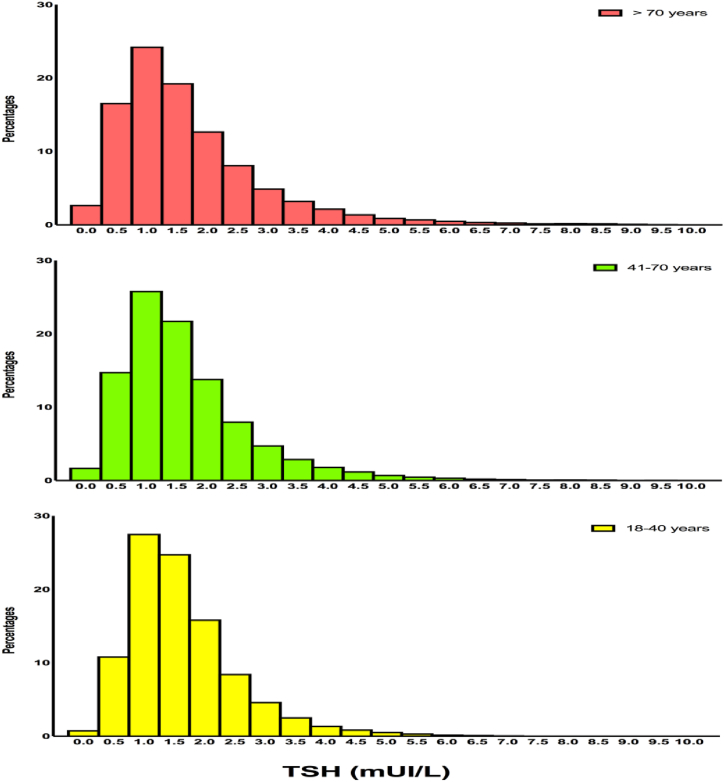
TSH distribution in the 3 age categories (18–40, 41–70, and >70 years).

**Fig. 3:**
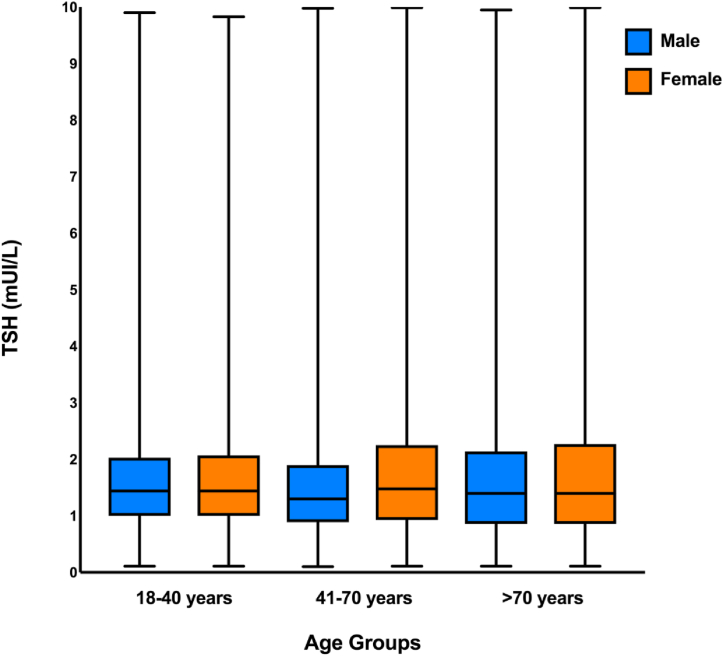
Cluster boxplot represents the median, minimum and maximum of TSH by age categories and by gender.

The TSH LRL decreased with the 7 age categories (p < 0.001) while the URL increased (p < 0.001). The TSH distribution according to the 3 age categories (18–40, 41–70, and >70 years) are depicted in Fig. 2. Similarly, the RI range became wider with increasing age: the LRL in the 3 age categories were 0.40, 0.30 and 0.23 mIU/L and for URL were 4.40, 5.00 and 5.71 mIU/L respectively. The median and interquartiles (first and third quartiles) of TSH values according to gender and age categories are illustrated in Fig. 3. No difference in the median TSH according to gender was noted in the age categories 18–40 (1.44 mIU/L for both genders, p = 0.18) and >70 (1.40 mIU/L for both genders, p = 0.23). However, in the age category 41–70, the median TSH was significantly higher in women compared to men (1.48 vs 1.30 mIU/L respectively, p < 0.001). For all age groups, the women's RIs were wider than the men's ones: respectively [(0.38–4.50) vs (0.45–4.0), p < 0.001] for the age group 18–40, [(0.27–5.30) vs (0.34–4.40), p < 0.001] for the age group (41–70), and [(0.21–5.90) vs (0.26–5.30), p < 0.001] for the age group more than 70 years old.

### 3.3 Changes in RIs over the last decade

**Fig. 4:**
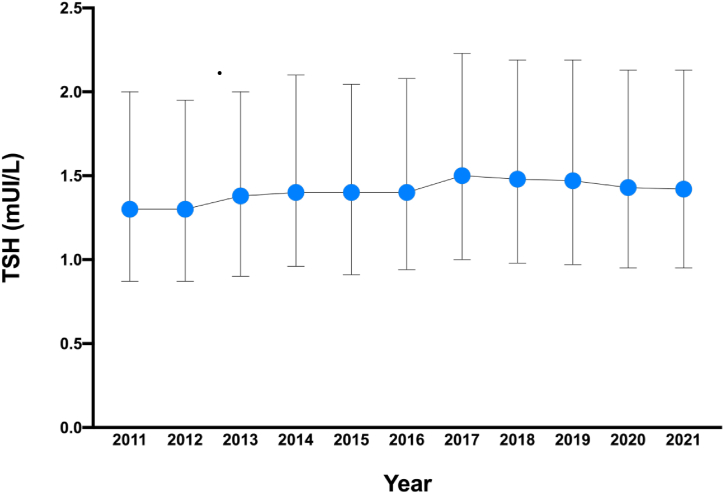
TSH distribution over the last decade (median and Interquartile range).

Table 2 and Fig. 4 show the median, 2.5th and 97.5th percentiles of TSH from 2011 to 2021 in the overall population and according to gender**.** In the overall population, the LRL and median TSH increased during the last decade from 0.28 in 2011 to 0.34 in 2021 (p < 0.001) for the LRL and from 1.30 in 2011 to 1.42 in 2021 (p < 0.001) for the median. On the contrary, the URL decreased from 5.10 in 2011 to 4.76 in 2021 (p < 0.001). This finding is observed even when the analysis is performed separately in men and women (p < 0.001 for the 3 comparisons of the LRL, URL and median in both gender). Table 3 shows the TSH RIs over the last decade according to age categories. There are significant differences in the median, LRL and URL across the years and in all age categories.

**Table 2:** Indirect reference ranges for TSH from the subjects’ date over the last decade and according to gender (reference values are given by 2.5th and 97.5th percentiles).

	N(%)	Percentile	2011	2012	2013	2014	2015	2016	2017	2018	2019	2020	2021	p-values
**Total population**	80156(100)	2.5	0.28	0.29	0.30	0.31	0.32	0.31	0.30	0.31	0.33	0.30	0.34	<0.001
		50	1.30	1.30	1.38	1.40	1.40	1.40	1.50	1.48	1.47	1.43	1.42	<0.001
		97.5	5.10	4.90	5.00	4.94	4.60	4.75	5.03	5.10	5.08	4.98	4.76	<0.001
**Women**	49733(62)	2.5	0.27	0.27	0.30	0.30	0.30	0.28	0.28	0.28	0.31	0.27	0.33	<0.001
		50	1.33	1.35	1.40	1.48	1.40	1.45	1.55	1.52	1.52	1.44	1.50	<0.001
		97.5	5.40	5.10	5.20	5.20	4.70	4.83	5.39	5.26	5.44	5.13	5.26	<0.001
**Men**	30423 (38)	2.5	0.30	0.33	0.34	0.35	0.35	0.34	0.35	0.38	0.35	0.34	0.34	<0.001
		50	1.30	1.25	1.30	1.40	1.30	1.36	1.44	1.42	1.40	1.40	1.32	<0.001
		97.5	4.46	4.54	4.39	4.40	4.40	4.45	4.59	4.44	4.60	4.59	4.26	<0.001

Data are expressed as median and 2.5th and 97.5th percentiles. To assess the statistical difference between groups, ANOVA test was used for p2.5 and p97.5, whereas the Kruskall-Wallis test was used for the medians.

**Table 3:** RIs for TSH from the subjects’ date over the last decade according to age categories (reference values are given by 2.5th and 97.5th percentiles).

		N(%)		2011	2012	2013	2014	2015	2016	2017	2018	2019	2020	2021	p-values
**Age categories**	18–30	11909(14.9)	2.5	0.37	0.41	0.45	0.50	0.45	0.42	0.46	0.46	0.46	0.43	0.39	<0.001
			50	1.32	1.3	1.40	1.50	1.50	1.50	1.54	1.59	1.51	1.45	1.43	<0.001
			97.5	4.09	3.85	4.33	4.30	4.11	4.45	4.37	4.85	4.86	4.29	3.75	<0.001
	31–40	12363 (15.4)	2.5	0.32	0.35	0.40	0.36	0.39	0.34	0.39	0.39	0.37	0.44	0.52	<0.001
			50	1.30	1.30	1.30	1.40	1.40	1.40	1.57	1.54	1.53	1.45	1.49	<0.001
			97.5	4.62	4.18	4.10	4.65	4.20	4.37	4.52	4.60	4.99	4.18	4.51	<0.001
	41–50	15134 (18.9)	2.5	0.32	0.30	0.34	0.36	0.33	0.30	0.34	0.32	0.32	0.35	0.33	<0.001
			50	1.36	1.40	1.40	1.48	1.40	1.40	1.51	1.45	1.49	1.50	1.41	<0.001
			97.5	4.80	5.30	5.02	4.53	4.40	4.62	4.79	4.64	4.59	5.41	4.91	<0.001
	51–60	15694 (19.6)	2.5	0.26	0.25	0.28	0.28	0.30	0.29	0.30	0.31	0.32	0.25	0.35	<0.001
			50	1.30	1.30	1.33	1.44	1.40	1.40	1.50	1.45	1.44	1.38	1.38	<0.001
			97.5	5.80	4.72	5.30	5.60	4.60	5.07	5.58	5.10	5.32	4.79	4.34	<0.001
	61–70	12177 (15.2)	2.5	0.22	0.26	0.24	0.27	0.30	0.26	0.29	0.30	0.30	0.27	0.34	<0.001
			50	1.30	1.22	1.30	1.35	1.35	1.36	1.40	1.41	1.41	1.38	1.32	<0.001
			97.5	5.05	5.10	5.01	5.20	4.70	4.95	5.22	5.26	5.31	5.66	5.13	<0.001
	71–80	8846 (11.0)	2.5	0.22	0.23	0.30	0.25	0.17	0.26	0.20	0.22	0.27	0.21	0.24	<0.001
			50	1.30	1.30	1.3	1.40	1.30	1.40	1.46	1.37	1.37	1.33	1.44	0.051
			97.5	5.49	5.50	5.33	5.59	5.50	4.70	5.66	5.76	5.73	5.75	5.92	<0.001
	>80	4033 (5.0)	2.5	0.26	0.19	0.21	0.23	0.30	0.22	0.22	0.20	0.29	0.22	0.21	<0.001
			50	1.42	1.32	1.40	1.50	1.56	1.50	1.60	1.52	1.47	1.66	1.47	0.009
			97.5	6.06	6.28	6.06	6.76	6.43	6.26	6.04	6.89	5.54	6.72	5.57	<0.001

### 3.4 Comparison of the RIs between the indirect and direct methods

A comparative analysis was conducted to evaluate the TSH reference range in the Lebanese population using the direct method [22] and the findings from the current study, which utilized the indirect method. It is important to note that these two studies were conducted on distinct populations. There was no significant difference in the median TSH between the indirect and the direct method in the overall population (respectively 1.40 and 1.36, p = 0.37), as well as in men (1.37 vs 1.41 respectively, p = 0.59) and in women (1.45 vs 1.35 respectively p = 0.08) (Table 4). However, in the overall population, the LRL was lower in the current study in comparison with the direct method (0.31 vs 0.53, p < 0.001, respectively). This finding was found in both men and women (for women 0.29 vs 0.51, p < 0.001, for men 0.35 vs 0.62, p < 0.001). Finally, the URL was higher in the current study in comparison with the direct method in the overall population (4.95 vs 4.09, p < 0.001, respectively) and in women (5.20 vs 3.61, p < 0.001) whereas no difference was seen for men (4.48 vs 4.63, respectively p = 0.54).

**Table 4:** Comparison of TSH data obtained from direct and indirect method.

		Indirect method	Direct method [23]	P-value
**Subjects** **Number (%)**	**Total population**	80 156 (100)	262(100)	
	**Women**	49733 (62)	166 (63)	0.66^a^
	**Men**	30423 [38]	96 [37]	
**Median TSH (mUI/L)**	**Total population**	1.40	1.36	0.37
	**Women**	1.45	1.35	0.08
	**Men**	1.37	1.41	0.59
**TSH 2.5th percentile (mUI/L)**	**Total population**	0.31	0.53	<0.001
	**Women**	0.29	0.51	<0.001
	**Men**	0.35	0.62	<0.001
**TSH 97.5th percentile (mUI/L)**	**Total population**	4.95	4.09	<0.001
	**Women**	5.20	3.61	<0.001
	**Men**	4.48	4.63	0.54

Indirect method obtained from data performed Immulite 2000 (Siemens, Healthcare Diagnostics, NY, USA, Diagnostic Products Corporation).

Direct method obtained from data performed on Cobas e411 (Roche Diagnostics, Mannheim, Germany).

a The p-value represents the comparison of sex ratio between the two samples of individuals (direct vs indirect).

## 4 Discussion

Our study established for the first time the TSH RIs of the Lebanese population using the indirect method from data collected over the last decade. This method is based on the analysis of the available database without the need for new sample collection. We showed that the TSH LRL and URL RIs were respectively 0.31 and 4.95 mIU/L. This RI was wider than the one provided by the manufacturer, which was 0.4–4 mIU/L.

In the literature, RIs for TSH vary in a way that LRL ranges from 0.17 to 0.64 mIU/L and URL ranges from 4.23 to 5.95 mUI/L [[24], [25], [26], [27], [28]]. This variation can be explained by ethnic disparities [29,30], changes in iodine status [11,30], different approaches to establish RIs (direct *vs* indirect), variability of the inclusion and exclusion criteria adopted in each study [13] or finally due to different assays [27]. Moreover, these discrepancies can be attributed to differences in methodology and sample size between studies. Three previous studies [25,31,32], using the indirect method, have shown that TSH RIs were not concordant with those of the manufacturer. In the first two ones, performed on Arab [25] and Chinese populations [32] the RIs were wider while, in the third one [31], performed on a Turkish population the RI was narrower. Conversely, the RI found in another Turkish study [28] was concordant with the manufacturer one. Consequently, each laboratory should establish its own TSH RIs and not rely on the manufacturer's one to make appropriate diagnostic decisions.

In women, compared to men, the median TSH was higher (respectively 1.45 vs 1.37 mIU/L), and the RI was wider (for the LRL 0.28 vs 0.35 and for the URL 5.2 vs 3.48). The median of TSH values in the current study were in concordance with several other studies [9,22,33]. Furthermore, a metanalysis performed in Europe [13] showed that median TSH was higher and reference ranges were broader in women compared to men. Similar results were seen in Italy with the median TSH values being 1.46 for women and 1.39 for men respectively [9]. In contrast, Vadiveloo et al. [12] showed in the Scottish population a significant higher median TSH levels in men but without clinical relevance. Moreover, two other studies [8,11] reported that RIs were wider in men in comparison to women. The higher median TSH and the wider RI in women compared to men can be explained by the higher incidence of autoimmune thyroid dysfunction in women knowing that in our current study, thyroid antibodies status was not included among exclusion criteria. On the other hand, women are more likely to experience TSH variability due to factors such as the use of thyroid replacement therapy for thyroid-related conditions or fluctuations in TSH levels during pregnancy. Therefore, the establishment of a sex-specific RIs for TSH for each population is crucial to allow a more accurate diagnosis and follow up of thyroid diseases.

In addition, our results showed that median TSH increased with age, a finding that was observed in both genders. Contradictory results were reported in the literature regarding the variation of median TSH with age. In the NHANES study [10] which included 16533 subjects, the median TSH increased with increasing age. Similarly, Zou et al. [22] reported in the Chinese population, an increase in median TSH in both genders with increasing age. However, other studies reported that the median TSH either gradually decreases with age in both genders [11,33] or that no obvious correlation was seen between serum TSH levels and age [8,14,28,34,35]. When evaluating RI in relation to age, our results showed that, in both men and women, the LRL decreased while the URL increased. In fact, the 2.5th percentile decreased by 0.20 mIU/L while the 97.5th percentile increased by 1.78 mIU/L, when comparing the age group 18–30 vs the one >80 years. Previous studies [[36], [37], [38]] demonstrated a similar increase in serum TSH URL with age. Similar findings were also observed in two additional studies from Scotland [12] and Turkey [39] where a wider RI was observed for elderly subjects. The progressive increase in TSH levels with aging was attributed to the increased prevalence of positive thyroid antibodies [10,40]. An age-related alteration in the TSH set point or a reduction in TSH bioactivity rather than the development of occult thyroid disease are also possible explanatory factors [41]. On the other hand, the lower LRL in elderly was related to the higher prevalence of subclinical hyperthyroidism probably in relation to the improvement in iodine supplementation over time [42]. Thus, adopting age specific RIs in elderly could avoid overestimating subclinical hypothyroidism or hyperthyroidism in this age group.

We then studied the trend of TSH values over the last decade. Our results showed that LRL tended to increase while the URL tended to decrease between 2011 and 2021. This finding was observed in the overall population as well as in both genders. This progressive decline in the URL over the last decade was detailed by Biondi [43] in his editorial. It might be explained by the increased sensitivity of TSH assays, by the development of more accurate thyroid antibody tests, or to a more accurate selection of the reference population. However, another recent German study [11] using the direct method has shown that there is a shift of the LRL from 0.25 mIU/l to 0.49 mIU/l and of the URL from 2.12 mIU/l to 3.29 mIU/l over a 10 years period. This finding was attributed to an improved iodine supply in Germany. In fact, excessive iodine intake can lead to iodine-induced hypothyroidism, through the persistence of the “Wolff-Chaikoff” effect, a finding that mainly occurs in individuals with previous autoimmune thyroiditis”[44]. On the contrary, Inal et al. [31] reported no differences between 2004 and 2008 for both LRL and URL TSH values, probably due to the short-term follow-up period of the study. In Lebanon, the first national survey conducted in 1993 revealed mild iodine deficiency among school-aged children. However, political instability in the country delayed the effective implementation of the Universal Salt Iodization (USI) program until 1995 [45]. Unfortunately, the efficacy of this program was found to be lacking in the 2013–2014 national survey, with a decrease in the median Urinary Iodine Concentration (UIC) of school-aged children to 66 μg/L [46]. Hence, worldwide, more studies are needed to evaluate the trend of TSH values through time.

We finally compared results of the current study to those obtained in our previous report [23]. In this report, using the direct method, the TSH RIs were determined on a sample of 262 healthy Lebanese adult subjects using the Cobas e411 Roche assay after strict exclusion criteria including: individual age less than18 or more than 65 years old; pregnancy, childbirth less than a year ago, or breastfeeding; current or previous hospitalization during the last month; history of thyroid disease or other chronic diseases; and intake of any drug that can affect thyroid tests. However, in the current study, that uses the indirect approach, only individuals with repeated TSH tests within the same year, those with a TSH levels below 0.1 mIU/L or above 10 mIU/L, and values from inpatients individuals were excluded to minimize the impact of thyroid disease on thyroid tests. Because the Immulite and the Roche assays are comparable, the RIs determined in both studies can be easily compared [47]. We found that the median TSH values were similar with no gender differences. However, both URL and LRL were significantly different in the overall population and in both genders, with wider RIs in the current study. The reason for that could be the inclusion of the elderly population, the non-exclusion of subjects with positive antibodies and/or a much larger number of TSH samples in the current study. Nevertheless, other studies reported a good concordance in TSH RIs between the direct and indirect methods [39,48]. Motor et al. [39] included the same age group population in both the direct and the indirect method which was not the case in our direct method study where subjects older than 65 were excluded. This finding can explain the differences in TSH RI between our two studies.

One limitation of our study was the non-applicability of the exclusion criteria (such as thyroid diseases, pregnancy or drugs mainly levothyroxine intake) that should have been considered in order to establish reliable TSH RIs [49]. Indirect methods offer substantial advantages, including efficiency and a reduced burden on patients, allowing for the use of existing data without the need for new health data collection. However, as described by Jones et al. [3], one limitation of the indirect approach is the potential influence of diseased subpopulations on the derived interval as well as the seasonal variation that may affect TSH RI [50]. Although our methodology did not explicitly exclude patients on specific medications, pregnant patients, or those with comorbidities, our study's stringent exclusion criteria effectively filtered out most patients with thyroid-related conditions. This was achieved by eliminating repeated TSH tests within the same year, as these typically indicate underlying health concerns. Additionally, we used outpatient clinic data to minimize the inclusion of individuals likely to have thyroid-affecting conditions and focused on patients with TSH levels between 0.1 and 10 to ensure a sample population with likely normal thyroid function.

Our study had many major strengths. The first one was the very large and representative number of TSH measurements, which increased the validity of our findings. Our hospital is also one of the largest tertiary centers in Greater Beirut, recruiting patients from all regions of Lebanon and thus ensuring a representative sample of TSH levels across the country. Second, the study included elderly subjects and allowed the establishment of RIs in this subgroup of the population. Third, the TSH measurements were performed in a single laboratory using the same reagent during the whole study period. Finally, in addition to age and gender, the trend of TSH RIs was also investigated over the last decade, a finding that was poorly studied in the literature.

It is worth mentioning that our study is the first to establish TSH reference intervals in the Lebanese population, using a large and robust database. This research not only demonstrates significant age and sex differences in TSH levels but also provides a foundation for more accurate interpretation of TSH values within this demographic. By recognizing these variations, our study supports a more tailored approach to diagnosing and managing thyroid diseases in Lebanon by using our own age and sex specific reference ranges. To build on this work, more collaboration is needed with other national hospitals to gather additional data and establish a comprehensive set of reference ranges across multiple institutions. The ultimate goal would be to standardize a single national reference range interval, replacing the use of manufacturer-provided ranges that do not account for our population's specific characteristics.

## 5 Conclusion

In conclusion, RIs for TSH in the Lebanese adult population were determined using the indirect method. Our RIs were wider than those obtained by the direct method and than those provided by the manufacturer. Therefore, our RIs are probably more suitable for physicians to diagnose and follow-up thyroid dysfunction disorders compared to the manufacturer ones. In addition, different age and gender RIs were established and should prompt the adoption of specific RI according to age and gender. Finally, because of the observed change over time in the TSH RI in our population, a periodic reevaluation of RI in every population should be recommended to assess the variability of TSH RIs with age, gender, ethnicity across the years.

## CRediT authorship contribution statement

**Elie Naous:** Writing – review & editing, Writing – original draft, Data curation. **Riam Hijazi:** Writing – original draft, Data curation. **Marianne Chalhoub:** Software, Methodology. **Nada El Ghorayeb:** Writing – review & editing, Software. **Marie-Hélène Gannagé-Yared:** Writing – review & editing, Supervision, Project administration, Methodology, Formal analysis, Data curation, Conceptualization.

## Ethics approval and consent to participate

Research have been performed in accordance with the Declaration of Helsinki and was approved by the ethics committee of our hospital named “Comité d’éthique de l’Hôtel-Dieu de France” (reference CEHDF 1524). No informed consent was requested by our Ethics Committee of Hotel Dieu de France (CEHDF 1524) since the data collection was anonymous and retrospective.

## Consent for publication

Not applicable.

## Availability of data and materials

The datasets used and/or analyzed during the current study available from the corresponding author on reasonable request.

## Funding

Not Applicable.

## Declaration of competing interest

The authors declare that they have no known competing financial interests or personal relationships that could have appeared to influence the work reported in this paper.
